# Supplementary material for: Exploratory study evaluating the relationships between perinatal adversity, oxidative stress, and infant neurodevelopment across the first year of life
Source: PLOS Glob Public Health. 2023 Dec 28;3(12):e0001984. doi: 10.1371/journal.pgph.0001984 (PMC10754429; doi:10.1371/journal.pgph.0001984)
Supplement: Attachment — Submitted filename: PLOSGPHResponse to Reviewers.docx [file pgph.0001984.s001.docx]

University of Southern California Keck School of Medicine

4650 Sunset Boulevard, MS #31

Los Angeles, CA 90027

323-361-6393

kgateau@chla.usc.edu

## Dr. Lisa Miyako Noguchi

## Academic Editor

*PLOS Global Public Health*

October 15, 2023

Dear Dr. Lisa Miyako Noguchi

I would like to thank you all for taking the time to review my article, provide feedback and offer me with the opportunity to revise and resubmit. Enclosed you will find my revised article that I am submitting to *PLOS Global Public Health*. Below you will find your comments, my responses and changes made based on your recommendations.

Thank you for your consideration!

**Journal Requirements:**

1. We have noticed that you have a list of Supporting Information legends in your manuscript. However, there are no corresponding files uploaded to the submission. Please upload them as separate files with the item type 'Supporting Information'.

***Fig 1: Adapted mediation model of early childhood adversity, oxidative stress and neurocognitive development (33) & Fig 2. Mean 8-IsoF2a across the first year of life were initially uploaded as “Figures” described as “Fig 1” and “Fig 2”. They are uploaded again for your review labeled as figures labeled Fig1.tiff and Fig 2.tiff.***

2. We noticed you have some minor occurrence of overlapping text with the following previous publication, which needs to be addressed: <https://secure-web.cisco.com/1uGAPjw8KERlh8dwTOXDwN4RaI_COyztxTYOGDmVzDJR08mwxsTlsxNzfkyJKpQyZslS5N2KPnI2BZ6Rb4VDfo2EimPlEwwSrYQB9vXk7Dod3U6Vm-3RBWZoVJ9UhqUvos0Ti0MrYr_nvxydg9A1BPiScpMXqCXgA6uYiLZf2XsU0xLePFaryEbpDScro0rKja6tGw8pBIIzyTcvpPHJITaJzD46szpH5fDnVhRkEQVX0d3dy0-2KeUf10hS1LZ-UC5geWodFJ5ppRWfcN5EnRFGRhqXOXPKzpfY9MqRll3d2WqItDJ8wv9d09fW0FfUjhRlnCjJZ6OmnLCfRDP6Zb3NJvQtNmsNMx36EvwLFTV3-Ap8Ka-gi85Wn13VAPqzMs_U3dDCVoT8OpLPbKiZVQprAYz2eE_DUYpQuVlz5vVWGOzKG-31NFxzYbup2CsgkRBk0yfwFJZ3vSkApXPDEEA/https%3A%2F%2Fwww.researchgate.net%2Fpublication%2F332694118_Childhood_adversity_mental_health_and_oxidative_stress_A_pilot_study>

**Thank you for highlighting this point. After an extensive review and external screen using overlap software the following instances were noted:**

***Line 8: Title Page “University of Southern California Department of Pediatrics, Los Angeles, CA”. This is affiliation is consistent with authors from both manuscripts***

***Line 10 “Children’s Hospital Los Angeles”. This is affiliation is consistent with authors from both manuscripts***

***Lines 244-245: “gas chromatography/negative ion chemical”. This describes the exact methodology by which 8-IsoF2a are evaluated and the corresponding literature is cited.***

***Line 280: “mental health scores for” changed to “mental health scores seen in the mothers”.***

***Line 301 “a significant correlation between” this bridging statement unifies two distinct clauses in the results sections that report independent findings.***

***Line 411 “There are several limitations to” changed to “ there are many limitations to this study”***

***Lines 430-431 “potentially important relations between” changed to “possibly crucial associations between”***

***References: All of the following are overlapping references cited in both manuscripts***

1. ***Felitti VJ , Anda RF , Nordenberg D ,Williamson DF , Spitz AM , Edwards V , et al .***
2. ***Shonkoff JP, Garner AS***
3. ***Schiavone S, Jaquet V, Trabace L, Krause KH***
4. ***Mansur RB, Cunha GR, Asevedo E, Zugman A, Rios AC, Salum GA, et al.***
5. ***Boeck C, Koenig AM, Schury K, Geiger ML, Karabatsiakis A, Wilker S, et al.***
6. ***Black CN, Bot M, Scheffer PG, Cuijpers P, Penninx BW***
7. ***Matthews MA, Aschner JL, Stark AR, Moore PE, Slaughter JC, Steele S, et al.***

**Reviewer Comments:**

**Reviewer #1:** This research is quite relevant to many settings and particularly at these times when newer diagnoses come into existence, but at the same time, it has become relatively difficult to assign/ attribute neurocognitive dysfunction or other diseases, especially those in adults, to a particular cause. Thus, an insight is given to this particular area that can become a point of focus in research and clinical areas.

Generally, issues presented are well articulated, discussions flow are coherent and are in line with objectives and methods proposed.

The quality of figures however, is relatively poor and it is advisable to include clearer images/ figures that will aid the reading.

***Thank you for this comment. Both figures have been redone and uploaded for review.***

**Reviewer #2:** Thank you for the opportunity to review "Exploratory study evaluating the relationships between perinatal adversity, oxidative stress, and infant neurodevelopment across the first year of life." The article focuses on a sample of n=116 mother-infant dyads recruited from primary care practices in Boston and Los Angeles. The article explores the relationship between adversity experienced by women during pregnancy, F2-isoprostane levels in their blood and their infants’ urine, and infant neurocognitive development during various time points in the first year of the infants’ lives. The implications of this research are important, because having a reliable biomarker to predict which infants will benefit from greater neurocognitive supports, and greater family supports, could assist pediatricians and the greater child welfare system to direct resources at higher-risk infants and their families.

Introduction: the research questions, claims, and context are clearly explained.

Related research is clearly described and linked to this study.

To make the article accessible to wider audiences, consider clarifying the definition of “internalizing disorders” on page 6, line 30, such as by providing examples of such disorders.

***Added the following text to expand on internalizing disorders Lines 130-131 “(generalized anxiety disorder, depressive disorder, bulimia and anorexia nervosa, dysthymic disorder)”***

On page 6, lines 146-149, please elaborate on what the existing literature says about the screening window to study the effect of maternal stress on neurocognitive development in offspring, and how it relates to the window chosen for your research study. What evidence is there that the chosen screening window is optimal?

***The following text was added to expand upon the importance of the perinatal period as a screening period Lines 151-158: “A review done by Van Den Bergh et al. reported on seventeen studies that evaluated the relationship between maternal perinatal stress and neurodevelopmental outcomes (41). This review found that over all perinatal stress was associated with cognitive, behavioral and emotional problems in infants, preschool and adolescent children. Work done by Silvera and colleagues evaluated cumulative perinatal adversity’s association with developmental outcomes. This study found that higher perinatal adversity scores were significantly associated with lower neurodevelopmental and school readiness scores, and increased behavioral problems (43). These important findings not only infer a crucial perinatal screening period they also highlight the long-lasting effects experiencing early adversity has on neurodevelopmental trajectories.”***

Methods:

On page 9, lines 226-230, consider mentioning what the maximum and minimum score are for the MSEL (minus the gross motor score, as described), for reference.

***The published reference for the early learning composite score (ELC) is the mean and standard deviation. The following language was added for context. Lines 231-232: “and has a mean of 100 and standard deviation of 15.”***

On page 10, line 235, consider mentioning the factors that can affect F2-isoprostane levels in urine and whether those were accounted for or not, or state that concentration is the only known factor, if appropriate.

***To date there is no consistent data on exogenous or endogenous factors that affect F2-isoprostane levels in the urine aside from the pathophysiologic processes that induce oxidative stress. Given the inconsistency of the published literature we did not adjust for any factors that have been shown to alter F2-Isoprostane levels in the urine. Added the following language to provide context for what has been shown:***

***Lines 240-241 “No other exogenous or endogenous factors have been consistently shown to effect urinary 8-IsoF2a levels thus no other adjustments were made.”***

Results and data:

Consider elaborating on your interpretation of why higher infant 8-IsoF2a at 2 months is correlated with a lower infant MSEL at 12 moths, but the opposite is true for infant 8-IsoF2a at 6 months and MSEL at 12 months.

***This is a very good question and something our group has had significant discussion about. The only positive correlation seen (statistically significant or otherwise) between 8-IsoF2a and MSEL at 12 months is with the infant 8-IsoF2a levels at 6 months. All other correlations are negative as to be expected. Additionally, we only saw statistically significant correlations with the 12-month MSEL as to be expected given the phenotypic effects of adversity are usually delayed and measurable at later time points. There is no compelling evidence for this being a true physiological phenomenon. This lone finding may be in part due to the 6-month time point being the physiologic nadir of oxidative stress and given most infants had lower levels of 8-IsoF2a this finding may be driven by a few individuals. Ongoing studies by this group with larger more diverse sample sizes are likely to provide more information about these correlations. The following text was added to further expand on these results Lines 401-407 “while higher 2 month infant 8-IsoF2a were significantly correlated with lower MSEL scores at 12 months, higher 6 month infant 8-IsoF2a were significantly correlated with higher MSEL cores at 12 months. This lone finding may be in part due to the 6-month time point being the physiologic nadir of oxidative stress and given most infants had lower levels of 8-IsoF2a this finding may be driven by a few individuals. Ongoing studies by this group with larger more diverse sample sizes are likely to provide more information about these correlations.”***

Figures and tables: figures and tables are clearly presented.

An editorial note on table 3: there is an inconsistency in the formatting of the r’s and p’s in the table. Some are capitalized and other are not. The tables and figures are otherwise correctly labeled.

***Thank you for this note. The table formatting has been changed for consistency.***

Sincerely,

Kameelah Gateau, MD, MS, FAAP

Assistant Professor of Clinical Pediatrics

USC Keck School of Medicine

Attending Neonatologist

Fetal and Neonatal Institute

Division of Neonatology | Children’s Hospital of Los Angeles
